# Bioactive and Biodegradable Nanocomposites and Hybrid Biomaterials for Bone Regeneration

**DOI:** 10.3390/jfb3020432

**Published:** 2012-06-20

**Authors:** Bedilu A. Allo, Daniel O. Costa, S. Jeffrey Dixon, Kibret Mequanint, Amin S. Rizkalla

**Affiliations:** 1 Department of Chemical and Biochemical Engineering, The University of Western Ontario, London, Ontario N6A 5B9, Canada; Email: ballo@uwo.ca (B.A.D.); dcosta@uwo.ca (D.O.C.); kmequani@uwo.ca (K.M.); 2 Department of Physiology and Pharmacology, Schulich School of Medicine & Dentistry, The University of Western Ontario, London, Ontario N6A 5C1, Canada; Email: jeff.dixon@schulich.uwo.ca; 3 Biomaterials Science, Schulich School of Medicine & Dentistry, The University of Western Ontario, London, Ontario N6A 5C1, Canada

**Keywords:** bioactive glass, biodegradable polymers, bone regeneration, hydroxyapatite, organic-inorganic hybrid, nanocomposite

## Abstract

Strategies for bone tissue engineering and regeneration rely on bioactive scaffolds to mimic the natural extracellular matrix and act as templates onto which cells attach, multiply, migrate and function. Of particular interest are nanocomposites and organic-inorganic (O/I) hybrid biomaterials based on selective combinations of biodegradable polymers and bioactive inorganic materials. In this paper, we review the current state of bioactive and biodegradable nanocomposite and O/I hybrid biomaterials and their applications in bone regeneration. We focus specifically on nanocomposites based on nano-sized hydroxyapatite (HA) and bioactive glass (BG) fillers in combination with biodegradable polyesters and their hybrid counterparts. Topics include 3D scaffold design, materials that are widely used in bone regeneration, and recent trends in next generation biomaterials. We conclude with a perspective on the future application of nanocomposites and O/I hybrid biomaterials for regeneration of bone.

## 1. Introduction

Bone defects, ranging from small voids to large segmental defects are a prevalent and persistent problem in clinical orthopedics and dentistry. Bone defects arise from a variety of causes including fracture nonunion [1,2], dental and orthopedic implant fixation [2], trauma or tumour resection [1,3,4], periodontitis [5,6], and musculoskeletal disorders such as rheumatoid arthritis [7]. In these and other clinical circumstances, bone repair and regeneration can be accelerated using natural and synthetic bone grafts are desired to ensure rapid restoration of skeletal function. Furthermore, intervention is necessary to repair nonunions or critical size defects, which are intraosseous wounds of a size that do not heal by regeneration, or in which some pathologic process exists that prevents regeneration. In these cases, bone-graft materials are often required to provide an osteogenic response promoting the formation of new bone [1].

Current standard procedures for bone defect repair include autografts and allografts [8,9]. These are tissues harvested from one individual and implanted into the same or a different individual, respectively. Autografts such as those derived from aspirated bone marrow, cancellous or cortical bone, or vascularized grafts are osteogenic, osteoconductive, and osteoinductive and are considered the gold standard [10,11]. However, autografts are associated with high operating costs for harvesting the graft, limited availability, donor site morbidity and complications including infection, pain, and hematoma [8,9,12,13,14]. On the other hand, allografts are subject to cleaning and preparation processes designed to remove cells to minimize immune response. These processing techniques potentially reduce the osteoinductivity, osteoconductivity, and mechanical strength of the graft [8,10]. To overcome these limitations, significant effort has been devoted to the development of biomaterials as bone-graft substitutes that can augment or regenerate bone [8].

Regeneration of bone tissue requires: (1) an osteogenic signal; (2) host cells that will respond to the signal; (3) three-dimensional (3D) scaffold designed to support the growth of responsive host cells and permit the formation of extracellular matrix (ECM); (4) a vascularized host bed [8]. The scaffold serves as a space filling construct providing cell anchorage sites, structural cues, bioactive agents and/or growth factors, as well as inhibiting the formation of fibrous or bridging tissue (a consequence of the natural rapid repair sequence), while providing space for newly synthesized tissue and integration with surrounding host tissue [15,16]. Ideally the scaffold material not only provides mechanical stability to the individual cells, but also to the surrounding tissue prior to the synthesis of functioning new tissue [16]. Therefore, it is advantageous to match the mechanical properties of the scaffold material to that of the targeted tissue in order to withstand *in vivo* stress and loading [12,17].

Bone tissue engineering can be defined as the use of a scaffold to induce bone formation from the surrounding tissue *in vivo*, or act as a delivery template for implanted bone cells and/or tissue, which have been grown and expanded *in vitro* [8]. A number of different strategies exist for the tissue engineering of bone. Hutmacher [12] describes one common strategy, which is subdivided in to six phases: (1) fabrication of a bioresorbable scaffold; (2) seeding of osteoblasts into the scaffold in static culture; (3) growth of immature tissue in a dynamic environment (spinner flask); (4) growth of mature tissue in a physiologic environment (bioreactor); (5) surgical transplantation; (6) tissue-engineered transplant assimilation/remodeling. However, a range of different tissue engineering concepts, varying from acellular scaffolds to cellular/scaffold constructs, which are implanted with little or no *in vitro* culturing, have been studied in various situations including large animal models and clinical applications. In these studies, the animal/human body served as the bioreactor [8,18,19]

In order to promote bone healing, a scaffold construct must provide osteogenic, osteoconductive, and/or osteoinductive activity to the specific defect site [10]. In the case of noncritical size defects, which heal naturally, tissue engineering principles can be used to accelerate bone regeneration by providing a construct to support osteoblasts attachment and ECM synthesis to bridge the defect. For nonunions and defects of critical size, often the osteogenic response is insufficient to promote complete healing. As such, the scaffold must provide an enhanced response by including sufficient number of osteoblasts precursors and/or ideal concentrations of osteoinductive growth factors [9].

## 2. 3D Scaffold Design for Bone Regeneration

The main purpose of scaffolds for tissue regeneration is to provide a supportive and conductive construct for the formation of new tissue [15]. Brekke *et al.* [20] compiled a comprehensive list of the critical considerations during 3D scaffold design determined from an extensive literature review. As such, scaffold constructs are to be fabricated as 3D porous structures with appropriate pore size, porosity, and interconnectivity between pores, to allow for cell and tissue ingrowths [8,21]. Large surface area to volume ratio is desirable to promote cell ingrowths and appropriate cell density and distribution to induce vascularization of the construct from the surrounding tissue. Meanwhile, high porosity and interconnectivity are fundamental for sufficient diffusion of nutrients and oxygen and removal of metabolic wastes [11,21].

For bone tissue engineering, scaffold architecture should mimic that of cancellous bone, which is characterized by a random pore structure [20]. *In vitro*, osteogenesis is enhanced by lower porosity, which suppresses cell proliferation and promotes cell aggregation, however, *in vivo* higher porosity and pore size results in greater bone ingrowth [17]. Initially, a pore size of 100 μm was thought to be a minimum requirement due to cell size and migration, and diffusion issues. More recently, studies have identified a pore size in the range of 200–400 μm as optimal for cell and bone-tissue ingrowths, and sufficient vascularization [8,17,20,21]. For example, an *in vitro* and *in vivo* study [22] which tested poly(ε-caprolactone) (PCL) scaffolds with different range of pore sizes, showed both chondrocytes and osteoblasts preferred larger pore sizes in the range of 380–405 μm when cultured *in vitro*. In contrast, when implanted *in vivo* (cranial defects of rabbits), PCL scaffolds with a lower pore size ranging from 290–310 μm showed more new bone formation, which progressed further into the center of the scaffold.

In view of critical scaffold design parameters and their application in bone tissue engineering, a number of techniques have been investigated to fabricate 3D scaffolds with high porosity and surface area. The conventional methods for scaffold fabrication include drop-on-demand printing,[23] gas foaming [24,25,26], solvent casting/particulate leaching [22,27,28,29,30,31,32,33,34,35], precipitation casting [36], electrospinning [37,38], microsphere sintering, particulate leaching [27,34,39,40,41,42], freeze-drying [43] and a combination of these techniques.

## 3. Scaffold Material Selection

Since natural bone matrix is a composite of biological ceramic (hydroxyapatite) and polymer (collagen), it is not surprising that several synthetic and natural biomaterials based on natural/synthetic polymers, bioceramics and their composites, and hybrids have been used to prepare scaffolds for bone tissue engineering application [12,43,44,45,46]. The following section is intended to discuss some of the basic characteristics of these materials.

### 3.1. Biocompatible and Biodegradable Polymers

Various types of natural (collagen, poly hyaluronic acid, chitosan and alginate, *etc.*) [12,45,46,47], and synthetic, polymers (poly (glycolic acid) (PGA), poly (L-lactic acid) (PLA), PCL, *etc.*) [12,48] have been investigated for bone regeneration. Although the preliminary results are promising for naturally derived polymers [45,46], concerns about the feasibility of finding large amounts of materials needed for clinical applications has prompted researchers to explore the use of synthetic polymers. These materials can be easily manufactured into differing shapes and their physical and degradation properties can be tailored for specific application. The remarkable property of these polymers is their ability to support the mechanical needs for a wide variety of applications such as screws and fixation devices in orthopedics [49]. In particular investigators have concentrated on synthetic biodegradable polymers that are approved by the United States Federal Food and Drug Administration (FDA) as suture materials. These polymers are mainly poly (α-hydroxy esters) that are degraded by hydrolysis, which can be metabolized and excreted. The most common of these polymers are PGA, PLA, PCL and their co-polymers [8,44,50]. However, in spite of their wide application in tissue regeneration poly(α-hydroxy esters) have suboptimal biocompatibility due to the acidic degradation products. Furthermore, they also have limited strength and mechanical stability to match with the bone when made with large volume fractions of macro-porosity, which is a critical design consideration for tissue regenerative materials. In addition, they are not osteoconductive and do not directly bond to bone. The commonly used biocompatible and biodegradable synthetic polymers for bone tissue engineering applications are summarized in Table 1.

Particularly, scaffolds for tissue regeneration are required to be at the very least, capable of supporting cell attachment and provide sufficient mechanical strength to resist fractional forces produced by cells and contractile forces exerted by the natural healing process *in vivo* [16,17]. For bone tissue engineering, the defect must be shielded from intrusion of competing cell types and formation of non-osseous tissue such as scar tissue, which forms as a result of a rapid repair sequence and can be a site for failure [10,20]. The scaffold material should be biodegradable and bioresorbable, allowing for excretion of the initial foreign material and its degradation by products. Ideally, the scaffold degradation rate is expected to be in consort with, or lower than the remodeling rate of the tissue, under physiological loading [12,16]. Bone regeneration scaffolds are thought to maintain their physical and mechanical properties for 3–6 months with mass loss only to occur after [12,13,14,15,16,17,18] months [17]. The majority of degradable polymer systems undergo bulk degradation, which is highlighted by a two-stage degradation process [12]. Initially, biodegradation begins with slow reduction in viscosity and molecular weight of the polymer. The second stage results mass loss characterized by diffusion of molecular chains out of the bulk polymer, resulting in an accelerated degradation and resorption kinetics. The release of acidic by-products often associated with mass loss degradation of polymer systems could be a potential cause of inflammatory reactions [12].

**Table 1:** Physical, mechanical, and degradation properties of selected biodegradable polymers used as scaffolds [48,49,51,52,53,54].

	Melting Point (°C)	Glass Transition temperature (°C)	Tensile Modulus (GPa)	Degradation Time (months)	Degradation Products
Poly(L-lactic acid)	173–178	60–65	1.5–2.7	>24	L-lactic acid
Poly (D,L-lactic acid)	Amorphous	55–60	1.9	12–16	D,L-lactic acid
Poly (Glycolic acid)	225–230	35–40	5–7	3–4	Glycolic acid
Poly (ε-caprolactone)	58–63	–60	0.4–0.6	>24	Caproic acid
Poly (D,L-lactic-co-glycolic acid) (50/50)	Amorphous	50–55	1.4–2.8	3–6	D,L-lactic acid and glycolic acid
Poly (D,L-lactic-co-glycolic acid (85/15)	Amorphous	50–55	1.4–2.8	3–6	D,L-lactic acid and glycolic acid
Poly(D,L-lactic-co-glycolic acid) (90/10)	Amorphous	50–55	–	<3	D,L-lactic acid and glycolic acid

### 3.2. Bioceramics

#### 3.2.1. Calcium Phosphates

Calcium phosphates (CaP) are biocompatible, osteoconductive, and possess remarkable ability to bond directly to bone [55,56]. In particular HA, has attracted a great deal of attention in dental and orthopedic applications due to its similarity to the mineral phase of bone and teeth [57,58]. Synthetic HA powder can be produced by a variety of wet chemical methods and solid state reactions [5,59]. Wet precipitation represents a common commercial route for HA production where the drop-wise addition of phosphoric acid to a suspension of calcium hydroxide, or reactions between calcium nitrate and ammonium phosphate, both under alkaline conditions, results in a calcium deficient apatite precipitate [5,60,61]. Hydrolysis methods are also used to prepare HA, using acid calcium phosphates such as dicalcium phosphate dihydrate, octacalcium phosphate or dicalcium phosphate anhydrous [5]. Commercially available CaP, such as β-TCP, are also easily hydrolyzed to produce HA [62]. Sol-gel chemistry, involving the hydrolysis of phosphorous containing alkoxides and calcium salt and subsequent polycondensation, is a well-known and widely studied synthesis route. Advantages of sol-gel techniques include atomic level molecular level mixing providing a high degree of control over the composition and chemical homogeneity of the final product. However, production of crystalline HA powders from sol-gel synthesis typically require calcinations at elevated temperature, which is associated with the formation of secondary phases such as β-TCP and granular particle shapes [63,64]. Alternatively, hydrothermal processes synthesize crystalline HA at relatively low temperatures (<250 °C) by subjecting calcium and phosphorus precursors to high pressure steam in an acid digestion bomb [65,66]. Recently, HA nanowires with tunable aspect ratio were synthesized by a combination of sol-gel chemistry and hydrothermal treatment in aqueous solvent [67].

The synthesis of compact and dense HA and TCP scaffolds for bone regeneration often requires high temperature sintering and are poorly degradable in their highly crystalline form, while their amorphous counterparts are mechanically too fragile to be used for fabrication of highly porous scaffolds. The dissolution rate for calcium phosphates is in the following order:

Amorphous HA > α-TCP > β-TCP > crystalline HA

the crystalline HA, which is sintered at high temperature, has high chemical stability in contact with tissue fluids, which leads to limited bioactivity and osteoconductive effect [68]. Alternatively, their amorphous counterpart are characterized by a high dissolution rate *in vivo*, which accelerates material desorption and elicit immunologic response. Consequently, the dissolution rate and subsequent bioactivity has been improved by synthesizing biphasic calcium phosphates (BCP) consisting of varying mixtures of HA and the more soluble β-TCP. BCP in the form of granules, blocks, and specifically designed shapes are commercially available and are used in numerous orthopedic and dental applications [69].

*In vitro* studies using human bone marrow cells showed improved cellular attachment, proliferation and differentiation when cultured on HA as compared to other commonly used biomaterials, titanium and high molecular weight polyethylene [70]. *In vitro* culturing of osteoblasts-like cells on to porous PCL scaffolds showed significant increase in osteoconductivity and bone formation when embedded with HA particles or coated with biomimetic HA [71]. Osteoconductivity is clearly evident from *in vivo* experiments. Improved bone ingrowths into porous implant materials was obtained when coated with CaP [72,73,74,75,76,77,78,79], as well as eliminating fibrous tissue encapsulation commonly seen at the tissue/material interface of implanted polymer scaffolds [80,81]. Indeed clinical applications of calcium phosphate coatings for total joint arthroplasty has shown improved osseointegration at bone/implant interface resulting in superior implant stability [82]. Further, *in vivo* studies have shown potential osteoinductivity of biomimetic CaP coatings where ectopic bone formation occurred when coated implants were inserted in nonosseous sites [83,84,85].

#### 3.2.2. Bioactive Glasses

Bioactive glasses (BG) are amorphous and biologically active silicate-based glasses. They can react with physiological fluids to form tenacious bonds to bone through the formation of bone-like HA layers when implanted into living tissue [86,87,88]. The bonding mechanism involves a sequence of reaction steps leading to the precipitation of a carbonated HA layer on the implant surface. Furthermore, these reactions, which lead to the release of critical concentrations of soluble ions, induce favourable intracellular and extracellular responses leading to rapid bone formation [89].

Early BGs were prepared by the classic quenching of melts comprising SiO_2_ and P_2_O_5_ as network formers and CaO and Na_2_O as network modifiers [86]. This was the route followed until the early 1990s when sol-gel processing was introduced for the synthesis of bioactive glasses [90]. The sol-gel synthesis consists of a series of hydrolysis and polycondensation reactions of metal alkoxides followed by ageing, drying and thermal stabilization. A metal alkoxide has the generic structure M-(OR)x, and is a molecule consisting of a central metallic ion (M) bound to functional organic groups (R) through an oxygen linkage (O). Metal alkoxides, such as tetraethoxysilane (TEOS) and tetramethoxysilane (TMOS) are often used as silica precursors due to their ability to readily react with water. The acid catalyzed hydrolysis reaction results in the replacement of alkoxy side chains with hydroxyl groups. Hydrolysis occurs through a nucleophilic attack on the silicon atom by the oxygen atom in the water molecule [91].

Hydrolysis: M-(OR)_4_ + 4(H_2_O) 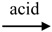 HO-M(OR)_3_ + R-OH

where –R represents an alkoxy functional group, e.g., C_2_H_5_OH.

The ratios of the reagents can be adjusted to control the degree of hydrolysis, ultimately leading to the formation of either clusters or branched polymeric chains. Subsequently, the polycondensation reaction results an increase in viscosity as the interconnectivity of the inorganic network grows [90,91]. These reactions are illustrated as follows:

Condensation: (OR)_3_M-OR + HO-/m(OR)_3_ → (OR)_3_M-O-M(OR)_3_ + ROH

HO-M(OR)3 + HO-M(OR)3 → (OR)_3_M-O-M(OR)_3_ + H_2_O

The condensation reaction liberates alcohol and water as a by-product. The water remains in the pores of the gel. The aging process holds water in the pores, enabling localized solution and reprecipitation of the solid network. This increases the thickness of interparticle necks and increases the density and strength. The aging process usually takes place for several hours/days at elevated temperatures [92]. The pore liquid and residual alcohol is removed from the monolith in the drying stage, leaving small interconnected pores with diameter in the range of 1–20 nm [92]. Stabilization at increased temperatures results further drying and removing of surface silanol groups and formation of three membered silica rings from the network. The process also increases density, strength and hardness and converts the glass network to resemble that of the melt-derived counterpart [90,93]. Addition of reagents such as tri-ethylphosphate (TEP) and calcium chloride or calcium nitrate yield the oxides of phosphorous and calcium, respectively.

The sol-gel route allows glasses of higher purity and homogeneity to be obtained, and the ranges of their compositions and textural properties to be expanded. In addition, all the steps in this route are carried out at temperatures notably lower than those required to obtain glasses by the melting method [90,94,95]. Therefore, it was no longer necessary to include components intended to decrease the melting temperature (*i.e.*, Na_2_O). In addition, the sol-gel-derived BG tends to have more simple compositions than melt-derived BG and due to the mesoporous structure the sol-gel derived BG exhibited enhanced bioactivity and resorbability. Furthermore, the presence of large number of silanol groups in the external surface of silica network enables the organic modification of the scaffold. Consequently, to prepare organic-inorganic hybrid scaffolds, which are chemically grafted with different active agents, such as certain peptides, proteins and growth factors, that act as powerful osteoinductive signals able to promote the appropriate bone cellular functions in the place where needed.

Most of the current studies on BGs are not only focusing on bone bonding, but also on their osteogenic potential and applications in bone regeneration. In addition to precipitating bone mineral, BGs have also been found to support enzyme activity [96], vascularization [97] as well as promote osteoblasts adhesion, growth and differentiation [98,99] BGs were also shown to induce the differentiation of mesenchymal cells into osteoblasts and to provide osteoconductivity [100]. The dissolution products of BGs have shown to exert a genetic control over osteoblast cycle and rapid expression of genes that regulate osteogenesis and the production of growth factor [101]. Silicon has been found to play a key role in bone mineralization and gene activation [102], which has led to the substitution of silicon for calcium in synthetic HA. *In vivo* investigation has shown that bone ingrowths into silicon-substituted HA granules was remarkably greater than that of pure HA [103]. Despite their advantages, BGs are much more brittle than natural bone, thus making them unsuitable for load bearing applications. Investigation of new strategies to enhance their mechanical property has been one of the main research interests. Coating of BG on organic polymer substrates or producing a composite of BG with organic polymer has been developed to “mimic” the composite nature of natural bone [95].

## 4. Emerging Biomaterials: Nanocomposites and Organic-Inorganic Hybrids

Despite the availability of materials with appropriate biological and structural properties they still need improvement to satisfy all the requirements for bone regeneration. A major stumbling block in the development of tissue engineering scaffolds is that most materials are not mechanically competent, bioactive and biodegradable all at the same time. Typically, mechanically strong materials are bioinert [104], while bioactive and biodegradable materials tend to be mechanically weak, when produced with large volume fraction of porosity [104,105]. Therefore, combining biodegradability, bioactivity and mechanical competence, hybrid and nanocomposite materials offer an exceptional opportunity to produce scaffolds with desired biological, physical and structural properties. O/I hybrid biomaterials differ from their nanocomposite counterparts wherein the inorganic components and the polymer chains interact through chemical bonding at the molecular level. Furthermore, O/I hybrids form a single phase material, consisting of a homogenous mixture between the organic and inorganic components. As such, the intimate nature of the organic-inorganic interface in O/I hybrids results in superior mechanical properties. 

**Figure 1:**
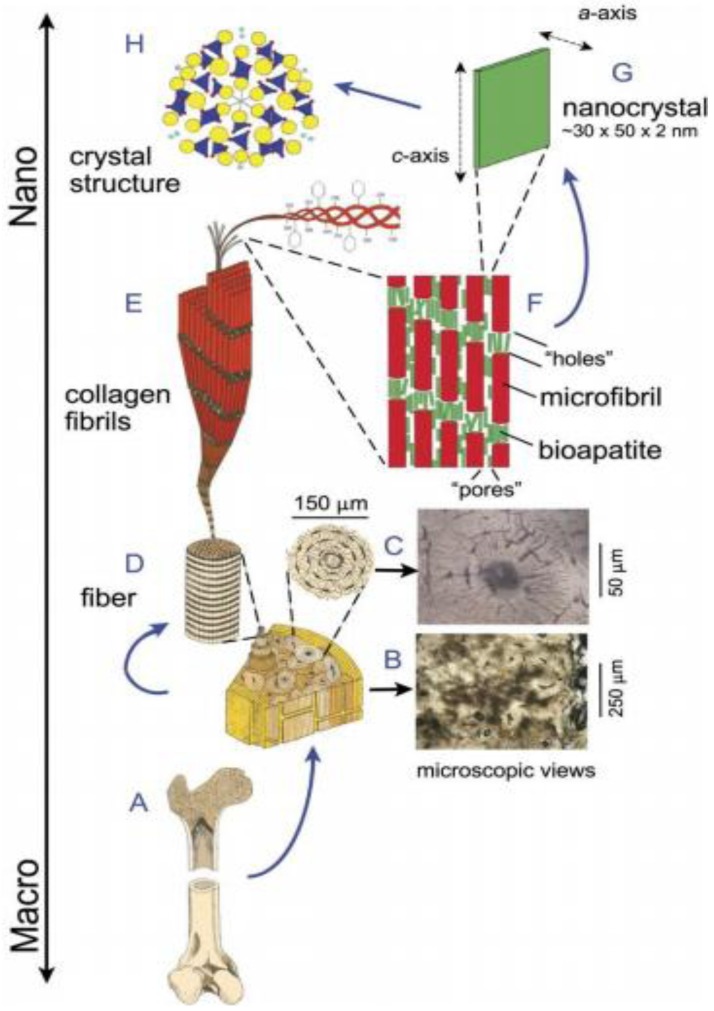
The hierarchical levels of typical cortical bone. (**A**) A longitudinal section of long bone; (**B**) Enlargement of a cross-sectional slice of cortical bone. Most of the volume of mature cortical bone consists of cylindrical osteons. Photomicrograph shows a thin-section of cortical bone with numerous osteons; (**C**) Enlargement of one osteon, consisting of a central vascular cavity with concentric lamellae. The black elliptical spots are osteocyte lacunae. Photomicrograph shows a single osteon; (**D**) One collagen fiber, created by the bundling of hundreds of fibrils, forms the structural framework of bone. Evenly spaced, dark bands represent periodic gaps (*i.e.*, “holes” seen in F) that occur between the ends of collagen fibrils laid down in overlapping arrays; (**E**) The smallest unit of the organic component in bone is the triple-helix collagen molecule. Five collagen molecules are bundled side by side in a staggered array, forming a microfibril; (**F**) Microfibrils, in turn, are bundled into fibrils E, F Enlargement of collagen microfibrils. Note that apatite crystallites (not to scale) form in voids. Each microfibril is ~300 nm long and ~4 nm thick; (**G**) An individual platelet of bioapatite. Unlike HA and FA, which crystallize into elongated prisms, biological apatite forms platelets, which are only about 2–3 unit cells thick; (**H**) View of the atomic structure of HA (as a stand-in for compositionally more-complex, less-symmetric bioapatite), viewed down the *c*-axis. For clarity, only the first couple of layers of atoms are shown, with PO_4_ groups indicated by tetrahedra. Yellow = calcium atoms; red = oxygen; dark blue = phosphate tetrahedra; light blue = hydroxyl. Reprinted from Reference [113] with permission.

From a biological perspective, the constituents of O/I hybrids and nanocomposites resemble the structure of bone tissue, where the inorganic component mimics the carbonated HA and the polymer component mimics the collagen rich ECM (Figure 1). Biodegradable polymers and bioceramics that have the ability to degrade *in vivo*, are ideal candidates for composite scaffolds, which gradually degrade while new tissue is formed. The release of acidic degradation by-products from polymers can cause inflammatory reactions, while the basic degradation of CaP or BG could buffer the acidic by-products. This may help avoid the formation of an unfavourable environment for cells due to the low pH.

Mechanically, bioceramics are stronger than polymers and play a critical role in providing mechanical stability to constructs prior to formation of new bone. However, most bioceramics are very fragile and prone to catastrophic failure due to their intrinsic brittleness and flaw sensitivity. The synthesis of O/I hybrids and nanocomposites capitalizes on the advantages of both material types. Increasing the content of the inorganic filler is generally proportional to an increase in stiffness. Typically, nanoparticles are highly aggregated and incompatible with the organic polymer matrix. This leads to an increase in the number of interfaces, which may give rise to more fracture surfaces resulting in crack propagation. Therefore, in order to optimize the mechanical properties of nanocomposites the surface of inorganic nanoparticles has been modified by grafting with organic molecules, which promotes polymer/inorganic-nanofiller compatibility and nanoparticle dispersion [106].

Nanocomposite materials can be prepared by adding inorganic nanoparticles or nanofibres into different polymer matrices. The size of the filler particles is an important parameter. The nano-sized fillers have a large surface area as compared to conventional (micro-sized) fillers. Nano-sized fillers can form a more tight interface with polymer matrix in composites, and hence, a high performance in mechanical properties is expected [107]. Furthermore, the intrinsic properties of nano-sized fillers contribute towards the different interactions between the filler particles and the polymer matrix. This leads to an increase in the mechanical strength and stiffness of composites in comparison to the properties of the unfilled polymer and of composites with micron-size reinforcement [108,109]. In particular, the particle size [110,111] and morphology [112] have measureable influences on the ability of HA to reinforce materials, with smaller diameters and larger aspect ratios (length/diameter) having the most profound effect on mechanical properties.

The increased specific surface area of nanoparticles showed an improved bioactivity compared to micron-sized particles. Webster *et al.* [114] have reported that a significant increase in protein adsorption and osteoblast adhesion has also been observed on nano-scale ceramic materials compared to micron-sized ceramic materials and composites. In related study, the bioactivity, degradation rate and mechanical properties of PLGA doped with nano-scale amorphous CaPs were strongly improved when compared to the pure polymer [115]. However, problems associated with poor interfacial bonding and particle agglomeration may be more pronounced when using nano-sized particles. As it is highlighted in the following sections, different strategies have been employed to improve the interfacial interaction between inorganic particles and polymer matrix, including silane coupling agents and polymer grafting on the surface of inorganic fillers. Recent studies [116,117,118] have also indicated that a sol-gel method can also be used to produce organic-inorganic hybrid materials with tailorable mechanical properties, controlled degradation profile and improved interfacial bonding between the inorganic and organic phase.

The following review sections are divided into two separate and distinguishable classes of biomaterials: (1) nanocomposites, either (i) conventional or (ii) surface modified, consisting of BG or HA as inorganic fillers in polymer matrices; (2) sol-gel derived O/I hybrids subdivided into (i) class I and (ii) class II hybrids.

### 4.1. HA Based Nanocomposites

Earlier studies have shown that HA powders consisting of micron-sized particles were successful in improving the mechanical performance of high-density polyethylene based materials [110,119], silk based porous scaffolds [120] and calcium phosphate cements [121]. The emergence of nanotechnology, coupled with the need for bioactive and biodegradable synthetic biomaterials has lead to the use of HA powders consisting of nano-sized particles, rods, and wires for producing nanocomposites for bone regeneration.

#### 4.1.1. Conventional HA-Based Nanocomposites

A host of different research groups have combined nano-sized HA with synthetic biodegradable polymers to produce nanocomposites for bone tissue engineering [122,123,124,125,126]. HA nanowires, having aspect ratios in the range 60–100, were used to produce dense nanocomposites comprising PCL as the matrix [67]. Mechanical testing of nanocomposites showed an increase in Young’s and compressive moduli. Scanning electron microscopy and energy dispersive X-ray spectroscopy of nanocomposites demonstrated a uniform distribution of HA nanowires within PCL [67].

Highly porous PLLA nanocomposite scaffolds were prepared by a thermally induced phase separation technique [122]. Unfilled PLLA and HA/PLLA nanocomposites scaffolds with greater than 89% porosity and pores sizes ranging from 50–100 μm were produced. Compressive modulus of nanocomposites scaffolds were significantly higher (8.3 MPa) as compared to unfilled PLLA (4.3 MPa). Scaffolds with varying HA content were immersed in fetal bovine/phosphate buffer solution to evaluate protein adsorption, which is thought to be a determining factor for cell adhesion and survival. Composite scaffolds containing high HA loading adsorbed 2–3 times more serum proteins than unfilled PLLA scaffolds. The authors believed the higher HA loading was more effective in protein adsorption because the increased HA weight fraction allowed for more HA particles to be exposed on the surfaces of the pore walls. The authors further showed that for high HA loading, composite scaffolds containing nano HA improved protein adsorption compared to scaffolds synthesized with micron sized HA particles at similar loading rates. 

Using a salt leaching and phase inversion process, Biossard *et al.* [123] developed porous nanocomposites scaffolds composed of biocompatible poly(ester urethane) (PU) and PCL with HA nano-particles. Micro-CT scans of scaffolds showed that scaffold pore size and porosity decreased with an increase in HA content, while the wall strut thickness increased. Results from the tensile test indicated that the Young’s modulus moderately increased for the nanocomposites compared with those without HA. At higher filler contents the HA particles aggregate, which may hinder the mobility of the polymer matrix. The authors concluded that preventing reorientation and alignment of the polymer segments, led to the formation of stress concentrations ultimately resulting in a decrease in the Young’s modulus of the composites. However, the authors did not address whether the improvement in mechanical properties of the composite was due to reinforcement of the polymer matrix by the HA filler, or by the decrease in porosity and increase in strut thickness as measured by the micro-CT analysis. 

PU/HA nanocomposites were further evaluated *in vitro* by a protein adsorption study and *in vivo* by a mouse dorsal skin fold chamber model to assess the biocompatibility and vascularization of biomaterials [127]. The nanocomposite and the unfilled PU scaffolds adsorbed protein on their surfaces, however the nanocomposite scaffolds greater levels of adsorption. Moreover, the *in vivo* results demonstrated that the host tissue response to the scaffolds were comparable for the PU/HA nanocomposites and the unfilled PU. The scaffolds promoted only a weak angiogenic host response, however, showed favorable biocompatibility with little acute leukocytic inflammatory activity throughout the entire study period.

Prabhakaran *et al.* [128] fabricated nanofibrous PLLA and PLLA/collagen/HA nanocomposite scaffolds, containing HA nanoparticles, by electrospinning. *In vitro* experiments, using cultures of human fetal osteoblasts, showed that the inclusion of HA nanoparticles in nanocomposites scaffolds enhanced cell proliferation, differentiation, and mineralization.

#### 4.1.2. Surface modified HA-Based Nanocomposites

Grafting of biodegradable polymers on the surface of nano-sized HA represents a unique approach to obtaining biomimetic nanocomposites materials for bone regeneration. The rationale of surface grafting is to improve the interfacial interaction between the organic and inorganic phases of the nanocomposites. The surface hydroxyl functional groups found on HA nano particles offer reactive groups for grafting with naturally derived chitosan [129] and the facile ring opening polymerization of various lactone based polymers such as poly-l-lactic acid (PLLA) and polycaprolactone (PCL) [130].

Hong *et al.* [131] developed a method of grafting PLLA on the surface nano HA crystals,ring-opening polymerization of L-lactide monomers in the presence of nano HA crystals (diameters of 20–40 nm) using stannous octanoate (Sn(Oct)_2_) catalyst. The surface grafted nano HA showed distinctly improved dispersion in both chloroform and PLLA composites, as compared to un-grafted HA. The grafting effect on the mechanical properties of the PLLA/HA nanocomposite was evaluated [131,132]. The results showed an increase in Young’s modulus with increasing filler content, however the difference was negligible. Improvement in mechanical strength of composites containing grafted HA was most notable by an increase in tensile strength. However, nanocomposites comprising un-grafted HA showed decreased tensile strength with an increase in filler content. Further improvements in mechanical properties were seen in the bending strength and modulus, and ductility of nanocomposites containing grafted HA. The stress-strain behavior of grafted HA composites resembled a tough material, exhibiting a necking phenomenon after yielding, in comparison to un-grafted composites, which displayed a brittle nature. The authors attributed the improvements in tensile strength and toughness of grafted HA composites to the grafted PLLA chains. The polymer chains on the HA surfaces, penetrate, entangle, and crystallize with the molecular chains of the PLLA matrix and therefore provide an interfacial covalent link. 

Wang *et al.* [133] evaluated the effects of PCL-grafted HA nanoparticles on the compressive modulus and strength of porous PCL/HA nanocomposite scaffolds produced by phase inversion/salt particulate leaching method. Results indicated a significant increase in both compressive strength and modulus with increase in filler content. Furthermore, the improvement in the mechanical properties was 50% higher for scaffolds containing surface grafted HA nanoparticles as compared to scaffolds with equivalent filler content of un-grafted HA.

Previous studies showed a limited reactivity with surface hydroxyl group of HA, which results in low polymer grafting density [130,131,134]. It has been proposed that increasing the grafting rate would improve the adhesion between the HA nanoparticles and polymer matrices. In an attempt to improve dispersion of HA nanoparticles in the nanocomposite and subsequently increase the mechanical properties, studies aimed at increasing the number of functional groups on the surface of HA nanoparticles, or reducing the steric hindrance allowing better access to hydroxyl groups have been reported. 

Qiu *et al*. [135] modified the surface of HA nanoparticles by grafting with L-lactic acid and followed by ring open polymerization. The chemical linkages were formed between calcium atoms on the surface of HA and the carboxylic groups of L-lactic acid and PLLA. In this study, the surface modification with L-lactic acid prior to the grafting process successfully increased the amount of grafted PLLA up to 22 wt%, which is significantly higher than previously reported values [130,131,134]. Tensile testing revealed significantly higher tensile modulus and strength of nanocomposites containing PLLA grafted HA as compared to nanocomposites consisting of pure HA. The surface modified HA with L-lactic acid imparted a toughening effect to nanocomposites, improving the ductility and allowing for an elongation up to 44% strain prior to fracture, in comparison to 12.4% and 6.5% for PLLA-grafted HA composites and unfilled PLLA respectively. The authors attributed the observed ductile behavior to a debonding of surface modified HA particles from the PLLA matrix, citing debonding in particle filled glassy polymers as a well-known phenomenon in producing yield points and ductile behavior. 

In a different approach, atom transfer radical polymerization (ATRP) was used to introduce new hydroxyl functional groups in the form of poly(hydroxyethyl methacrylate) (PHEMA), prior to ring-opening polymerization of caprolactone [134]. The PHEMA grafted HA nanoparticles was able to increase the grafting rate of PCL on HA nanoparticle surfaces to over 20 wt%. Similarly, ATRP was used to modify the surface of HA nanoparticles with poly(methyl methacrylate) [136]. The authors reported 48.7 wt% PMMA content on the HA surfaces and a large increase in water contact angle confirming the change from hydrophilicity of HA nanoparticles to hydrophobicity, which will lead to better compatibility with polymer matrices.

The applicability of the surface grafted HA in biomedical applications was evaluated by *in vitro* experiments using primary cultures of human chondrocyte cells [132]. Nanocomposite films containing PLLA-grafted HA showed higher levels of chondrocyte attachment and proliferation over 7 days, attributed to a reduction in the amount of lost HA during rapid degradation of nanocomposite films. A subsequent cell biocompatibility study using primary cultures of rabbit osteoblasts was conducted on nanocomposites produced of poly(lactide-co-glycolide) (PLGA) and PLLA-grafted HA nanoparticles to confirm the potential of the nanocomposites for bone regeneration [137]. An increase in cell attachment and proliferation was observed for films consisting of PLLA grafted HA as compared to unfilled PLLA films after 1, 3, and 5 days culture.

The findings from the vitro experiments prompted a further study by Zhang *et al.* [138] to evaluate *in vivo* mineralization and osteogenesis of porous unfilled PLGA and PLGA nanocomposites scaffolds containing (10 wt%) PLLA grafted and ungrafted HA. Scaffolds were implanted into dorsal muscles, and radius critical size defects in a rabbit model. The results showed improved osteoconductivity, mineralization and osteogenesis for scaffolds containing HA nanoparticles as compared to unfilled PLGA. The study revealed no decrease in osteogenesis or osteoconductivity of the HA due to surface grafting of PLLA, while providing improved HA particle distribution in the pores and better microstructure stability of the nanocomposite scaffold *in vivo*.

**Figure 2:**
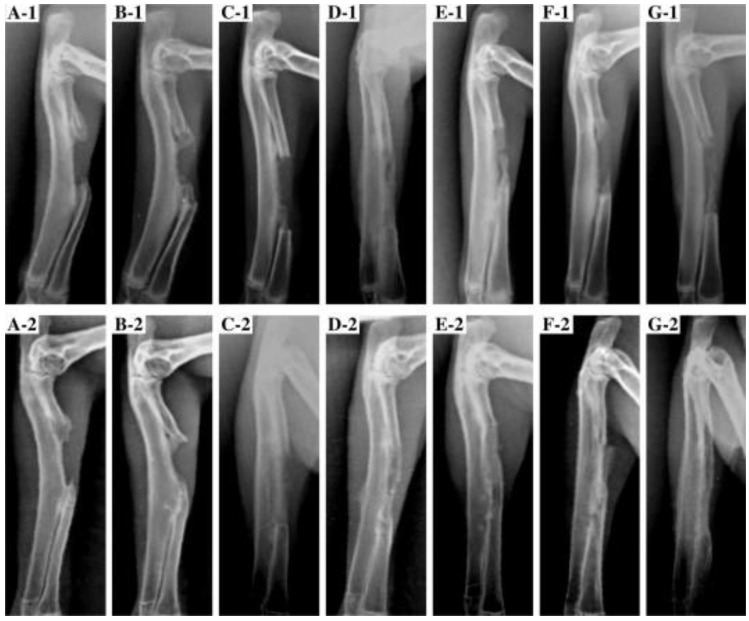
Typical radiographs of rabbit radius resection implanted with composites: untreated control (A-1,2), PLGA (B-1,2), 5 wt% PLLA-g-HA/PLGA (C-1,2), 10 wt% PLLA-g-HA/PLGA (D-1,2), 20 wt% PLLA-g-HA/PLGA (E-1,2), 40 wt% PLLA-g-HA/PLGA (F-1,2) and HA/PLGA (G-1,2) taken at 4 (1) and 24 (2) weeks post-surgery. Reprinted from Reference [139] with permission.

A similar rabbit radius critical size defect model was used to assess the ability of porous PLGA scaffolds, and PLGA nanocomposites containing 10 wt% HA, and 5–40 wt% PLLA-grafted HA, to repair the defect after 24 weeks post-surgery [139]. Radiographic evaluation (Figure 2) revealed differences in the defect healing, where the untreated and PLGA scaffold defects showed limited new bone formation, which was found exclusively at the ends of the defect, and was unable to bridge the gap. Meanwhile, PLGA nanocomposite scaffolds containing HA and PLLA-grafted HA, induced new bone formation that successfully bridged the defect. Furthermore, nanocomposites comprising 10 and 20 wt% grafted HA developed a greater calcified callus and were 2–3 times larger in volume compared to all other groups. Histologically micrographs further revealed that the defects treated with PLGA scaffolds were almost entirely filled with fibrous tissue. Conversely, defects treated with nanocomposites of 10 and 20 wt% grafted HA were filled with bone ossein, whereas only a small amount of bone ossein and capillaries were found in defects treated with nanocomposites containing ungrafted HA. It may be inferred that the use of grafted HA nanoparticles in the nanocomposites greatly improved the osteoconductivity of PLGA scaffolds. Specifically, the nanocomposites containing 10 and 20 wt% grafted HA induced the optimal healing of critical size defects by possessing the proper surface topography and roughness, pore size and porosity, degradation rate, and mechanical properties and stability that provided a more stable 3D construct for cell growth and extracellular matrix formation throughout the healing process.

### 4.2. Bioactive Glass Based Nanocomposites

#### 4.2.1. Conventional BG-Based Nanocomposites

Bioactive and biodegradable nanocomposites, which combine sol-gel derived BG nanoparticles/nanofibers and biodegradable polymers, have become very promising systems for bone regeneration because of their high osteoconductivity, osteoinductivity and biodegradability. They combine the strength and bioactivity of the BG and the ductility and toughness of the biodegradable polymers. In order to yield nanocomposites with high bioactivity and strong mechanical properties, various nanocomposites containing BG nanoparticles and biodegradable polymers were developed. Hong *et al.* [140,141,142] prepared a 3D porous PLLA/BG nanocomposite scaffolds containing different concentrations of sol-gel derived BG nanoparticles. Addition of BG nanoparticles up to 20 wt% did not alter the morphology of the scaffold. Whereas, the *in vitro* bioactivity study demonstrated that the scaffold containing 20 wt% had the best bone-like apatite forming ability. The compressive modulus of the PLLA/BG nanocomposite scaffolds increased from 5.5 to 8.0 MPa, while the compressive strength showed minor increase from 0.28 to 0.35 MPa as the BG content increased from 0 to 30 wt%. The inclusion of BG nanoparticles increased the water uptake of the nanocomposite scaffolds at lower BG content, in addition, greatly influenced the degradation rate of the PLLA matrix [140].

BG nanofibers (BGNF) prepared by electrospinning have also been used to prepare nanocomposites. The high surface area-to-volume ratio of nanofibers has been hypothesized to provide more cell attachment sites (and therefore a higher cell density per unit of space) compared with nanoparticles with lower aspect-ratio. Kim and co-workers developed well dispersed nanocomposites from PLA [143], collagen [144], PCL [145] matrices and a sol-gel-derived electrospun BGNF [146]. These nanocomposites showed good bioactivity, inducing HA precipitation on their surfaces when exposed to a simulated body fluid (SBF) [144]. It was also observed that the presence of BGNF in nanocomposites improved the osteoblast-like cells attachment, spreading and proliferation.

The effect of aspect ratio of the sol-gel derived BG fillers on the biocompatibility and mechanical properties of PCL/BG composites was investigated [108]. In this study, PCL/BGNF nanocomposites were compared with PCL micron-sized BG particle (BGp) composites. At 20 wt% filler content, the nanocomposites of BG nanofibers displayed significant improvement in both biological and mechanical properties as compared to composites with the micron-sized fillers. The results for the tensile test indicated that the elastic modulus of the PCL/BGNF nanocomposites was significantly higher than the PCL/BGp composites and the unfilled PCL. In addition, the nanocomposites of BGNF showed enhanced *in vitro* biocompatibility and osteoblast activity as compared to the PCL/BGp composites. Furthermore, *in vivo* animal test results revealed good biocompatibility and bone forming ability of the PCL/BGNF nanocomposite when implanted in a calvarial critical-size bone defect. In general, results from this study demonstrated the benefits of using fillers with high aspect ratio and surface area to volume ratio (*i.e.*, BGNF) instead on particulate filler in preparation of composite scaffolds. 

#### 4.2.2. Surface Modified BG-Based Nanocomposites

Surface modification of BG nanoparticles with biodegradable polymers represents a unique approach to improve the interface compatibility between the BG nanoparticles and the polymer matrix. In order to achieve this objective, a low-molecular weight PLLA was grafted to the surface of BG nanoparticles by using a diisocyanate coupling agent [147,148]. The enhanced interaction and adhesion between the grafted BG nanoparticles and the PLLA matrix resulted improvement in mechanical properties. At lower BG content, the grafted-BG/PLLA composites exhibited greater tensile strength than ungrafted-BG/PLLA composites. However, no significant difference in tensile modulus between grafted-BG/PLLA and ungrafted-BG/PLLA nanocomposites was observed. The morphology of the tensile fractured surface of the composite also showed that surface grafted BG nanoparticles were dispersed homogeneously in the PLLA matrix. The *in vitro* studies also revealed that the addition of nanoparticles improved the bioactivity of nanocomposite scaffolds [107].

### 4.3. Sol-Gel Derived Organic-Inorganic Hybrids

Organic-Inorganic hybrid materials can be either homogeneous systems derived from monomers and miscible organic and inorganic components, or heterogeneous systems (nanocomposites) where at least one of the components’ domains has a dimension ranging from some Å to several nanometers [149]. Aside from the intrinsic physical properties of the components, hybrid materials can also display special new properties as a result of the nature and degree of interfacial interaction between the two components. Since the traditional processing conditions for inorganic materials usually involve high temperature, it precludes the incorporation of organic compounds. Thus, the low temperature synthesis of sol-gel process allows it to be well adopted for the preparation of organic-inorganic hybrid materials and have proven to be effective [150]. The intimate molecular mixing promotes the organic and inorganic components to form a hybrid with small grain sizes and large interfaces [150]. These interactions result in a new material, with tailorable mechanical, chemical, and physical properties depending on the desired application [149]. The chemical reactivity of organic and inorganic species is usually quite different and phase separation tends to occur during the synthesis. Therefore, it is imperative that chemical bonds are formed between the organic and inorganic components in order to produce organic-inorganic hybrids. The nature of the interfacial chemical bond has been used to categorize these materials into two distinct classes. In class I, the organic and inorganic phases exchange weak interactions such as van der Waals and hydrogen bonds. In class II materials the two phases are linked through strong covalent bonds [149,150,151].

#### 4.3.1. Class I Sol-Gel Derived O/I Hybrids

Monolithic and porous O/I hybrids consisting of BG and water soluble polymers were prepared via sol-gel route. Martin *et al.* [118] incorporated poly(vinyl alcohol) (PVA) into the sol-gel synthesis of BG. The results of this study showed that the addition of polymer favored the synthesis of bioactive and crack-free O/I monoliths. However, an increase in PVA content resulted in O/I hybrid materials, which disintegrated when exposed to a buffer solution [118]. In other studies, up to 30 wt% PVA was incorporated to prepare PVA/BG hybrid foam scaffolds with interconnected pore networks and pore size of 500 μm [107,152]. The compression test showed that the strain at failure and compressive strength was increased for the PVA/BG hybrid as compared to pure BG foam. Conversely, lower compressive modulus was obtained for the PVA/BG hybrid foams as compared to the pure glass foam. The applicability of PVA/BG hybrid scaffolds towards bone regeneration could be limited because of two major reasons. First, PVA is not biodegradable and second, due to the weak hydrogen bond, which links PVA and BG, the O/I hybrid is likely to fail in a physiological environment [107,118,152].

#### 4.3.2. Class II Sol-Gel Derived O/I Hybrids

To overcome the limitations of water soluble polymer based hybrids, linking the polymer and inorganic phase by a strong chemical bond is imperative to improve the stability and performance under physiological conditions. For this purpose, coupling agents are used to functionalize the polymer to form a covalent bond with the inorganic phase and create a class II hybrid material. One of the widely studied sol-gel derived organic-inorganic hybrid biomaterials used Poly(dimethoxysilane) (PDMS) as a precursor [153,154]. These hybrids can be structurally described as a silica network covalently bonded to PDMS. However, the *in vitro* apatite formation ability of these hybrids was not satisfactory unless Ca^2+^ ions are incorporated in the network [155,156]. The hybrids show relatively large amount an apatite-like phase is deposited on their surfaces within only 12 to 24 h in SBF. From these studies, it was observed that the apatite formation ability is increased with the inorganic content, whereas PDMS provides better mechanical properties. In general, PDMS-derived hybrids show high ductility, however, their strength and Young’s modulus are much lower than those of natural bones. Although excellent coupling can be achieved, PDMS is not a degradable polymer. It is preferable to have a biocompatible and biodegradable polymer with a strong coupling potential. 

Biocompatible and biodegradable polymers have also been incorporated in attempt to prepare O/I hybrids. PCL/Silica hybrids were successfully synthesized via sol-gel process, in which PCL is intimately mixed into the silica network [157,158,159,160]. The silica network was achieved using 3-isocyanatopropyltriethoxysilane (IPTS) as the coupling agent in the presence of 1,4-diazabicyclo2.2.2octane. The coupling agent only reacts with the terminal hydroxyl groups; thus the amount of cross-linking in the hybrid is controlled by the molecular weight of the polymer [161]. To increase the cross-linking in this PCL hybrid, a reduction in the molecular weight of the polymer is required. Faster and more uniform nucleation and growth of apatite crystals was observed in the hybrid using lower molecular weight PCL. It was hypothesized that this behavior was mainly caused by the evenly distributed and well dispersed silica-rich domains, which acted as nucleation sites for the formation of the apatite crystals, and partly caused by the fast degradation of the PCL phase, which induced the fast release of calcium ion into SBF [151,162]. The PCL content in the hybrid system affected the bioactivity and mechanical properties of the PCL/silica hybrid material [117]. The higher PCL content in the hybrid resulted in lower apatite-forming rate and higher toughness. On the contrary, the lower PCL content in the hybrid exhibited higher apatite-forming rate and lower toughness. The highest values of tensile strength, Young’s modulus, and strain at failure were achieved in the hybrids with 60 wt% PCL content and were around 21 MPa, 600 MPa, and 50%, respectively [117]. These materials had tailorable bioactivity, degradability and mechanical properties, but the potential is limited by the coupling sites, which are at the end of the polymer chains. The lack of Ca^2+ ^ions in the hybrid system, which is essential in providing osteogenesis and improved bioactivity of the hybrid material, might also limit its potential application in bone tissue engineering. Experimentally, incorporation of Ca^2+^ in the hybrid system exhibited good osteoconductivity as hybrids are coated with bone-like apatite layer [163].

In fact, hybrid materials demonstrated some of the advantages of combining polymers with inorganic and bioactive materials. As many of the tissues within the body are nano-scale composites, it seems logical that this be considered when developing scaffold biomaterials for bone regeneration and repair. The ability to use a single phase or material for such purposes may be impractical, and composites may be utilized to yield better results. Such is the case with organic-inorganic hybrids, which can exhibit a range of bioactive, resorbable, and mechanical properties. Tailoring of material chemistry and morphology can thus be employed to match these properties with the host tissue, in an effort to give better incorporation and enhanced efficacy.

## 5. Conclusions and Future Prospects

**Figure 3:**
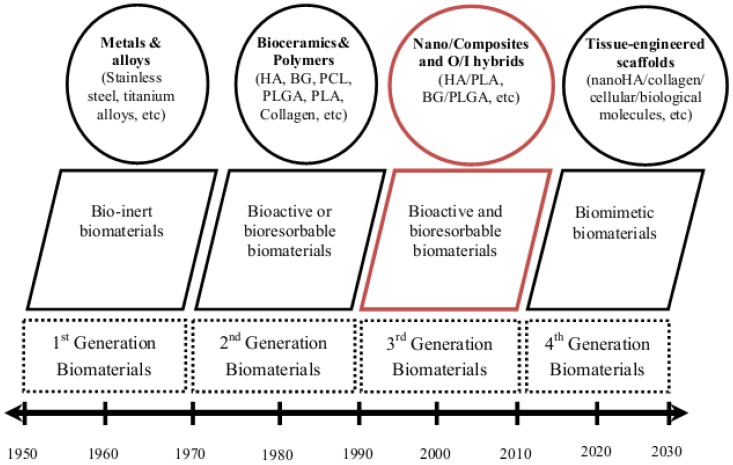
Evolution of biomaterials in bone regeneration and repair.Modified with permission from Reference [164,165].

The purpose of this article was to review the current state and challenges towards developing bioactive and biodegradable nanocomposite and O/I hybrid biomaterials, while highlighting the promising steps taken to improve the mechanical and biological properties for application in bone regeneration. Due to rapid advances made in the field, it was not possible to include all aspects of the work. However, every effort was made to ensure that seminal works and significant research findings are included, with minimal bias. The need for bone graft materials has led to the synthesis of various materials with different properties. The historical development of synthetic biomaterials for bone grafts with respect to their properties under physiologic environment has been classified into four generations (Figure 3). First generation biomaterials including stainless steel, cobalt, titanium and their alloys have a long history in dental and orthopedic applications, specifically for load bearing applications. The success of metal biomaterials is due, in part, to their resistance to corrosion, passive oxide layer, high strength, and good biological response. However, a mismatch in the stiffness of bone compared to the high stiffness of metals may lead to stress-shielding and subsequent implant loosening. Furthermore, metal biomaterials are not bioactive or biodegradable. Due to their combined bioactivity, biodegradability, and mechanical properties, bioactive and biodegradable scaffolds (3rd generation biomaterials) are becoming the focus of recent trends in biomaterial development for bone regeneration. The morphological (pore size and porosity), mechanical, and biological properties of bone, result in challenges for fabricating scaffolds suitable to regenerate large (critical size) cortical bone defects and capable of functioning under relevant loads. In view of this, as discussed in this review, various attempts have been made to exploit the novel properties of synthetic scaffolds with different morphologies for a variety of orthopedic applications. However, several issues need to be addressed prior to their clinical application, such as mechanical competence, biodegradability, and induction of vascularization.

The potential exists for scaffolds with tunable biological and mechanical properties to be achieved with nanocomposites and O/I hybrids materials. Bioactive and biodegradable nanocomposite or O/I hybrid scaffolds consisting of biodegradable polymers reinforced with bioceramics (BG or HA) phases are increasingly preferred for bone regeneration because they closely mimic the natural composite structure of bone. This resemblance in structure translates to improved cell response as compared to conventional composites, and depending upon factors such as materials and processing method, the mechanical properties may also be improved. As reviewed in the above sections, several research groups have produced nanocomposite and O/I hybrid biomaterials and scaffolds. The potential application in bone regeneration has been highlighted by assessing mechanical and biological properties (*in vitro* and *in vivo*). Selected works are summarized in Table 2.

**Table 2:** Bioactive and Biodegradable Nanocomposite and O/I Hybrid Biomaterials.

Material	Scaffold Fabrication Method	Mechanical Properties		*In vitro*	*In vivo*	Reference
		Modulus	Strength			
PLLA/HA	Phase separation, electrospinning	8.3 MPa (compressive)	3 MPa (tensile)	+	−	[122,128]
PLLA/collagen/HA	Electrospinning	-	2 MPa (tensile)	+	−	[128]
PU/PCL/HA	Salt leaching/phase separation	1.26 MPa (tensile)	-	+	+	[123,127]
PLLA/g-HA	Solvent casting (dense)	2.5–4 GPa (tensile)	58–75 MPa (tensile)	+	−	[131,132,135]
PCL/g-HA	Salt leaching/phase separation	0.75 MPa (compressive)	70 Pa (compressive)	−	−	[133]
PLGA/g-HA	Solvent casting (dense), salt leaching	3.7 MPa (tensile),	75 MPa (tensile), 2.31 MPa (compressive)	+	+	[137,138,139]
PLLA/BG	Thermally induced phase-separation	8 MPa (compressive)	0.35 MPa (compressive)	−	−	[140]
PLA/BG, PCL/BG	Electrospinning/thermal pressing (dense)	−	−	+	−	[143,145]
PLLA/g-BG	Solvent casting (dense)	3 GPa (tensile)	69.2 MPa (tension)	+	−	[147,148]
O/I Hybrid Monoliths	Sol-gel (dense)	600 MPa (tensile)	21 MPa (tensile)	−	−	[117,163]
O/I Hybrid Scaffolds	Sol-gel/Foaming	−	0.3 MPa (compressive)	−	−	[107,152]

**Figure 4:**
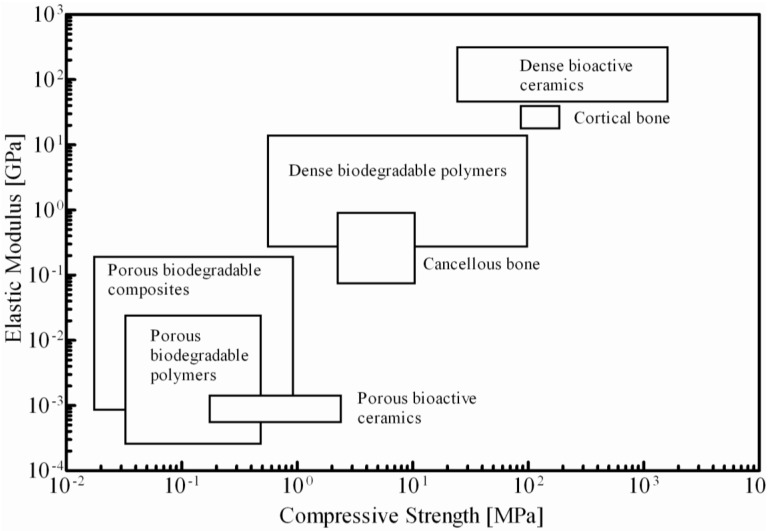
Elastic modulus *vs.* compressive strength of biodegradable polymers, bioactive ceramics and composites scaffolds with high porosity (>75%) and mostly interconnected pore structure. Modified with permission from References [104,105].

In order to use nanocomposites or hybrid scaffolds in a load-bearing application, the mechanical properties should approach to those of bone. The elastic modulus of cortical bone (in both transverse and longitudinal directions) is 6–23 GPa and its tensile strength ranges from 80 to 150 MPa [166,167]. In view of the present state of the art, the mechanical properties of bioactive and biodegradable porous scaffolds used for bone tissue engineering fall short of native bone (Figure 4). In lieu of this, improving the nanofiller dispersion via surface modification or grafting has been attempted to enhance the mechanical properties of nanocomposite scaffolds. Indeed, several of the nanocomposites reviewed in this paper fall within the range of the strength of bone. These results highlight the importance of structure-property relationship, particularly the importance of chemical structure and bonding, on the mechanical properties of nanocomposites. The combined effect of the polymer and the inorganic nanofillers contribute to the stiffness, strength and toughness of the resultant nanocomposite scaffolds. As with bone, the collagen of the ECM provides the intrinsic capability to deform to very large strains, while the HA nanocrystals provide the stiffness and resistance to deformation. As such the design of nanocomposite scaffolds based on biomimetics justifies the reinforcement of polymers with higher strength bioceramic nanofillers, to improve the stiffness. Therefore, choice of appropriate materials and improving the structure-property relationships are important components in scaffold design as the interfacial interaction between the nanofiller and the polymer matrix contribute significantly to the final mechanical properties and biological response.

In an effort to improve the interfacial interaction between the inorganic and the organic phase, and to reduce the domain size effect seen in conventional composites, sol-gel derived O/I hybrids has emerged as candidate biomaterials for 3D scaffold fabrication. The molecular level interaction between the inorganic and organic chains, which is observed in sol-gel derived O/I hybrids, has the potential to provide effective bone bonding ability with appropriate toughness and controlled degradation. The synthesis of O/I hybrids requires understanding of the atomic level interaction between the inorganic and organic components, and the distribution and cross-linking mechanism, as this will dictate the final properties of the resultant O/I hybrid. However, the challenges associated with the complexity of the synthesis procedure limits the progress of developing an ideal O/I hybrid scaffold for bone regeneration. In order to overcome this challenge, an optimal synthesis procedure should be developed through the collaboration between material scientists, synthetic chemists, biologist and clinicians.

As has been discussed, most of the current studies are focused on optimizing the scaffold properties in regards to the mechanical properties and bioactivity. However, development of bioactive and biodegradable 3D scaffolds with osteogenic and angiogenic potential remain a major challenge, because cells will not survive without an adequate blood supply. One of the alternatives to improve osteogenic and angiogenic potential of these materials is via the incorporation of active biomolecules such as growth factors into the scaffold structure. Surface modifications of nanocomposites or O/I hybrids through their surface functional groups may provide sites for a better cell attachment and responses. Recently, this strategy is becoming a promising trend for regulated *in situ* secretion/expression of angiogenic growth factors (e.g., vascular endothelial growth factor (VEGF)) and osteogenic markers (e.g., alkaline phosphatase) at therapeutic levels, which leads to successful vascularization and bone formation (mineralization) of scaffolds. However, there remains limited understanding regarding the long-term *in vivo* behavior of porous 3D nanocomposites and O/I hybrids scaffolds, particularly regarding the degradation mechanism, ion release kinetics and angiogenic stimulus of these highly porous systems. Future developments of scaffolds may need to consider the use of stem cell technology to obtain an ideal nanocomposite or O/I hybrid scaffolds for bone regeneration.
